# Using an adaptive modeling framework to identify avian influenza spillover risk at the wild-domestic interface

**DOI:** 10.1038/s41598-024-64912-w

**Published:** 2024-06-20

**Authors:** Diann J. Prosser, Cody M. Kent, Jeffery D. Sullivan, Kelly A. Patyk, Mary-Jane McCool, Mia Kim Torchetti, Kristina Lantz, Jennifer M. Mullinax

**Affiliations:** 1 U.S. Geological Survey, Eastern Ecological Science Center, Laurel, MD 20708 USA; 2 Volunteer to the U.S. Geological Survey, Eastern Ecological Science Center, Laurel, MD 20708 USA; 3 Department of Environmental Science and Technology, University of Maryland, College Park, MD 20742 USA; 4 Department of Biology, Frostburg State University, Frostburg, MD 21532 USA; 5 U.S. Department of Agriculture, Animal Plant and Health Inspection Service, Veterinary Services, Strategy and Policy, Center for Epidemiology and Animal Health, Fort Collins, CO 80521 USA; 6 National Veterinary Services Laboratories, Animal and Plant Health Inspection Service, USDA, Ames, IA 50010 USA

**Keywords:** Avian influenza, Waterfowl, Poultry, Spillover events, Wild to domestic interface, Influenza virus, Agroecology, Ecological epidemiology

## Abstract

The wild to domestic bird interface is an important nexus for emergence and transmission of highly pathogenic avian influenza (HPAI) viruses. Although the recent incursion of HPAI H5N1 Clade 2.3.4.4b into North America calls for emergency response and planning given the unprecedented scale, readily available data-driven models are lacking. Here, we provide high resolution spatial and temporal transmission risk models for the contiguous United States. Considering virus host ecology, we included weekly species-level wild waterfowl (Anatidae) abundance and endemic low pathogenic avian influenza virus prevalence metrics in combination with number of poultry farms per commodity type and relative biosecurity risks at two spatial scales: 3 km and county-level. Spillover risk varied across the annual cycle of waterfowl migration and some locations exhibited persistent risk throughout the year given higher poultry production. Validation using wild bird introduction events identified by phylogenetic analysis from 2022 to 2023 HPAI poultry outbreaks indicate strong model performance. The modular nature of our approach lends itself to building upon updated datasets under evolving conditions, testing hypothetical scenarios, or customizing results with proprietary data. This research demonstrates an adaptive approach for developing models to inform preparedness and response as novel outbreaks occur, viruses evolve, and additional data become available.

## Introduction

Wild waterfowl have long been recognized as reservoirs for avian influenza viruses (AIVs), with infection from low pathogenic avian influenza (LPAI) often yielding asymptomatic infection^1^. Although some strains of H5 and H7 LPAI possess the potential to rapidly become highly pathogenic (HPAI) during replication in gallinaceous poultry where there is high density, such occurrences typically present limited threat to wild bird hosts. However, the emergence of a specific lineage of H5 HPAI in the late 1990s led to an alteration of this paradigm, as this lineage (A/goose/Guangdong/1996-like [gsGD]) spilled over and subsequently was maintained in wild bird populations^2^. Following its emergence, gsGD H5 quickly spread through Asia, and once established in migratory wild bird populations, has led to several intercontinental events, causing notable losses to wild birds, agriculture, and even impacting human health. In the ensuing decades, gsGD H5 has become endemic in several countries in Africa, Asia, and along migratory routes in Europe^3^.

The gsGD H5 lineage presents an ongoing and continuously evolving threat to domestic poultry and wild birds at an intercontinental scale^4,5^. For instance, the incursion of gsGD H5N1 into North America in late 2021 has caused wild bird mortality events across the continental United States^6^, including over 40 large scale events (> 100 dead wild birds within an event;^7^). Furthermore, more than 60 million domestic poultry have died directly from infection or related control efforts through the course of this outbreak^8^. While there remains some uncertainty whether endemicity will occur within North America wild waterfowl^9^, the ongoing circulation of both gsGD H5 along with typical North American LPAI strains among wild birds signals ongoing risk of AIV transmission across the wild waterfowl—domestic poultry interface.

Due to the large economic impacts related to AIV outbreaks, there have been efforts to identify areas of transmission risk at the wild waterfowl—domestic poultry interface. Many of these efforts focus on farm-to-farm transmission^10–12^ or introductions to backyard farms^13^ without focus on initial introductions from wild birds to domestic poultry flocks. Despite identifying relationships between wild waterfowl abundance and outbreak risk^14–16^, many outbreak prediction efforts either do not include direct representation of wild waterfowl^17–19^ or have relatively static representations of their dynamic temporal distributions^20–22^. This lack of waterfowl inclusion is driven by limited availability of data on wild bird distributions and abundances^23^. While this issue persists for certain regions of the globe, the emergence of species-specific waterfowl abundance estimates at high spatial and temporal resolution for the entire continental United States^24,25^ presents an opportunity to advance AIV transmission risk models for this region.

**Figure 1:**
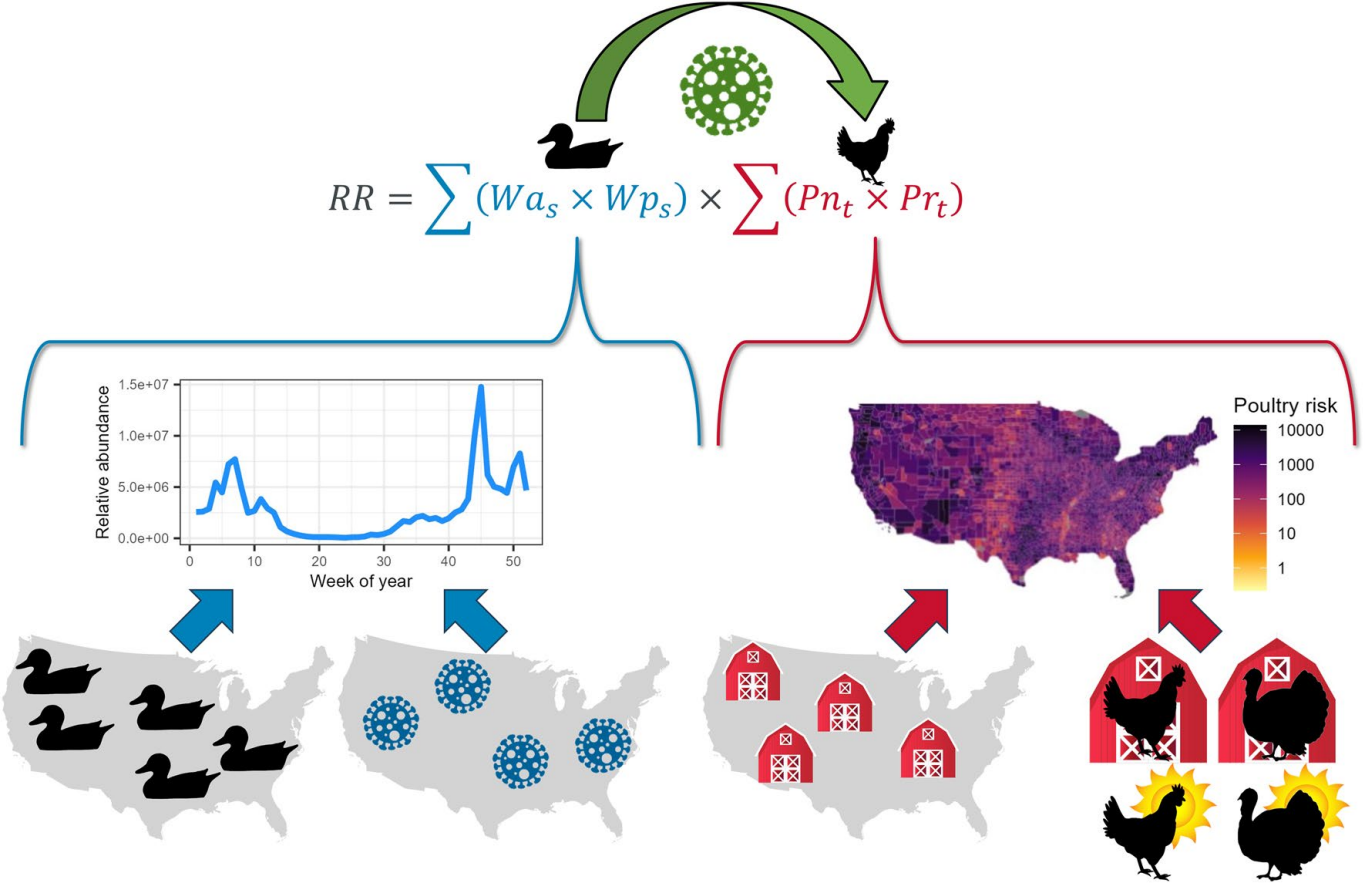
Graphical depiction of model components. The model seeks to quantify the risk of endemic avian influenza viruses spilling over from wild waterfowl reservoirs into domestic poultry operations. This risk can be measured as the product of the risk component associated with wild waterfowl and the risk component associated with poultry operations. For waterfowl, this component equals the relative abundance of infected waterfowl at a given time and place, which can be calculated as the relative abundance of waterfowl (*Wa*) multiplied by the proportion that are infected with AIV (*Wp*) for each species (*s*). For the poultry side of the equation, this risk component can be broken down into the number of poultry facilities (*Pn*) multiplied by the risk (*Pr*) of each type of poultry facility (*t*). The modular nature of this model allows for adaptive management as new data are collected and compiled, such as the inclusion of a wider variety of avian and non-avian wild hosts, hypothetical models of prevalence in the event of a novel incursion event, more detailed or proprietary locations of poultry facilities, and the inclusion of additional biosecurity information.

The objective of this work is the formation of a flexible model to estimate the risk of AIV transmission, which includes both highly and low pathogenic strains, from wild waterfowl to domestic poultry across the contiguous United States at a fine spatial and temporal resolution that can be easily adapted as new information is generated or under novel scenarios. Here, we sought to incorporate species-specific estimates of waterfowl abundance and AIV prevalence in a spatial and temporal context with domestic poultry distributions and relative risks dependent on production and poultry type. As data availability currently restricts the AIV prevalence models to endemically circulating viruses, development of these models is a first step towards providing adaptive tools to support decision makers in preparing for and responding to outbreak scenarios. Validating these models using phylogenetic analyses of poultry virus isolates to identify wild bird spillover events into farms from the current gsGD H5 clade 2.3.4.4b outbreak will assess the model’s ability to predict spillover locations for these novel incursions despite the initial use of currently available endemic virus prevalence models. We stress that these models, while designed to estimate the risk of endemic LPAI transmission into domestic poultry operations, can be readily altered for potential novel scenarios (Fig. 1).

## Results

### Overall results

**Figure 2:**
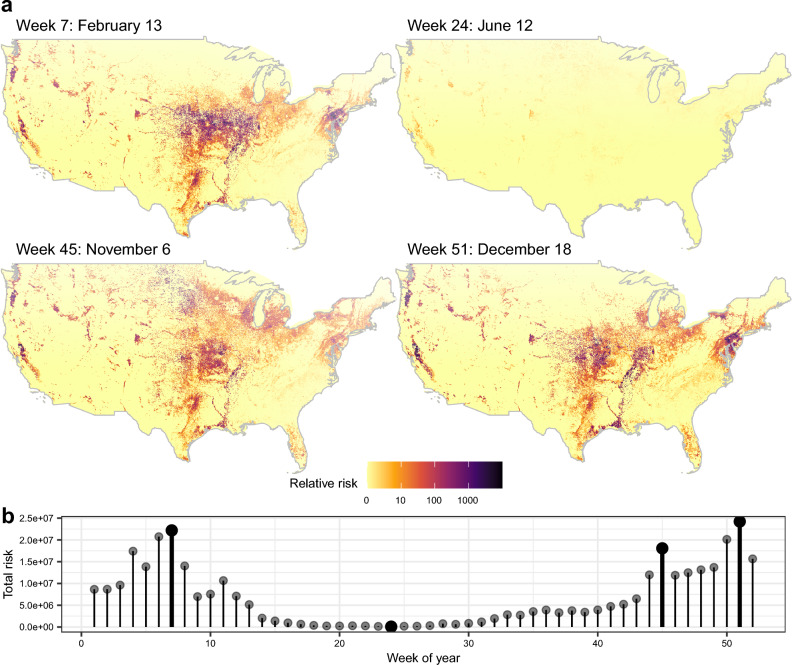
(**A**) Examples of weekly relative avian influenza transmission risk maps at the wild waterfowl and domestic poultry interface (3 km spatial and weekly temporal resolutions shown here; county level spatial resolution models were also run and are provided in released model outputs^26^). (**B**) Total relative risk across the entire country for each week of the year. Maps shown in A were chosen to display the peak levels of risk during autumn and spring waterfowl migration, the non-breeding season, and the low point in the summer. Dates highlighted in Panel **A** are bold in Panel **B**.

**Figure 3:**
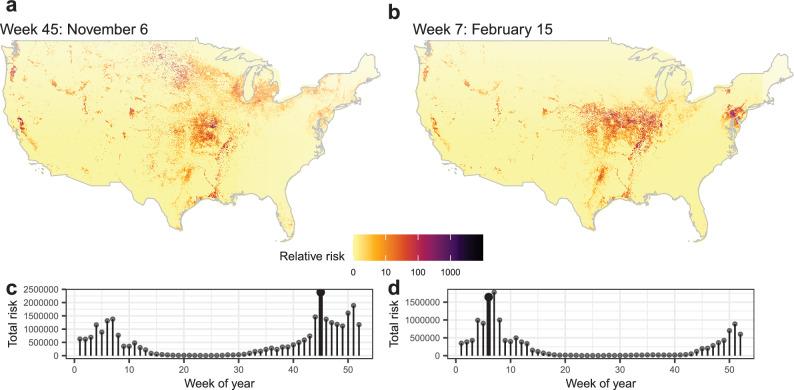
Examples of relative transmission risk maps for the H5 (Panels **A, C**) and H7 (Panels **B**, **D**) avian influenza viruses at the wild-domestic interface. (**A**) 3 km resolution spatial H5 relative risk map for the peak risk week 45 (approx. November 6) as seen in (**C**). (**B**) 3 km resolution spatial H7 relative risk map for peak week 6 as seen in (**D**).

**Figure 4:**
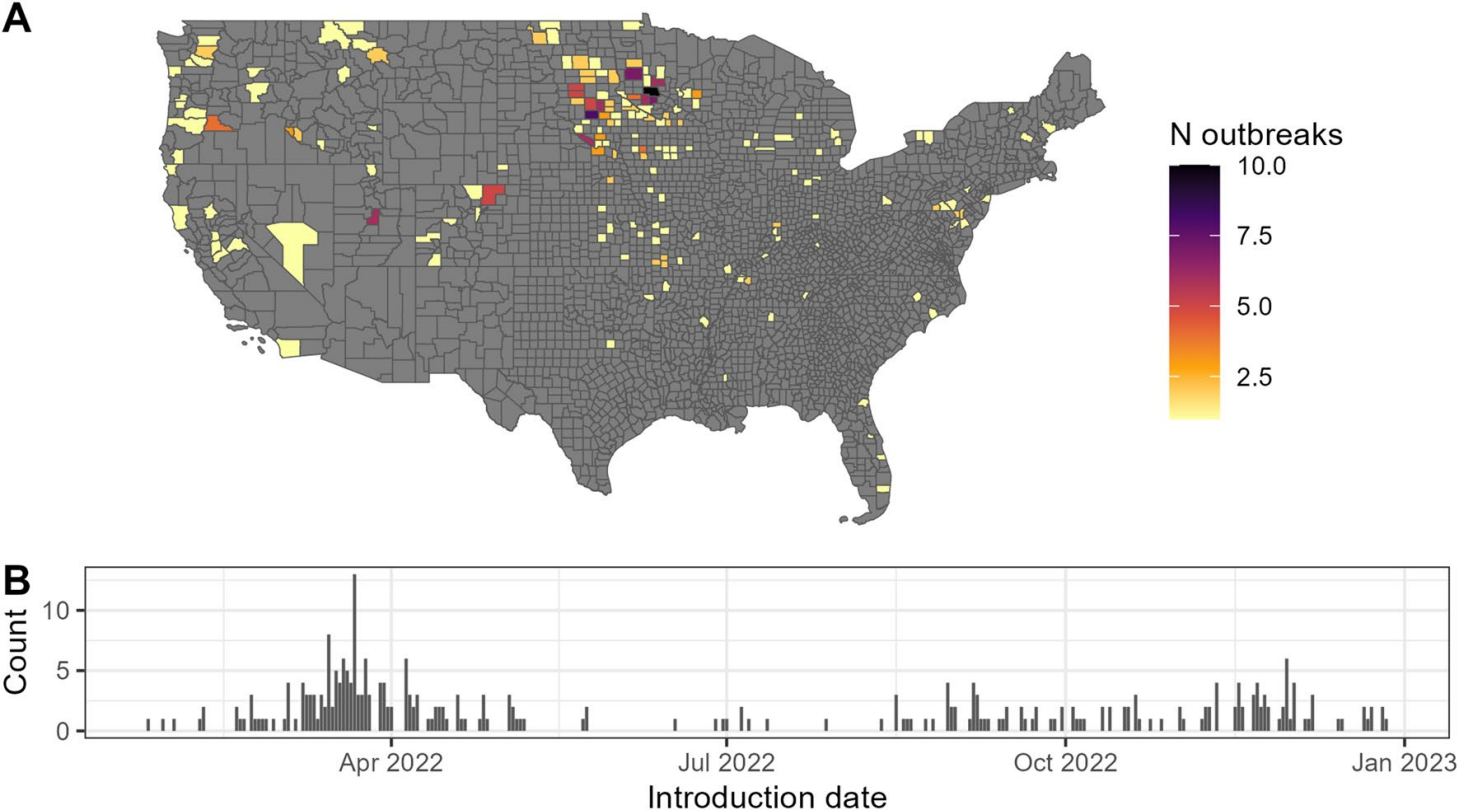
(**A**) Distribution of spillover events into domestic poultry farms during the 2022–2023 HPAI incursion mapped at the county level for the contiguous United States. (**B**) The temporal distribution of these introduction events, estimated to be two weeks prior to the confirmation of each positive flock, as identified by the WOAH^59^ 14-day incubation period preceding outbreak events*.*

Results for 4 model sets are provided as both fine-scale (3 km^2^ resolution) rasters, as well as summed to the county level for each week of the year (See Data Availability): (1) the primary model set using endemically circulating LPAI prevalence spatiotemporal model inputs, (2) models for H5 subtypes, (3) models for H7 subtypes, and (4) the primary model set that includes an environmental transmission component. Rasters for the model components are also available in the data release^26^. Figure 2 shows results for the primary model set, highlighting some of the larger, broad-scale seasonal differences. In general, we see the predicted risk of spillover events (of either highly or low pathogenic strains) shifting north and south with waterfowl migration, and the overall level of risk rising and falling with avian influenza prevalence, reaching its lowest levels in late spring and early summer. Additionally, we see some areas of particularly high poultry production that remain at an elevated risk year-round (i.e., risk at certain locations remains comparatively higher throughout the year than in surrounding areas, though relative risk does still increase and decrease throughout the year). Relative risk for the two subtype models showed stronger variation throughout the year, although with similar spatial patterns. Namely, the H5 model shows a more dramatic increase in risk during the late fall (Fig. 3), while the H7 model shows an increase primarily during spring (Fig. 4), consistent with when these two subtypes are most prevalent.

The version of the model that sought to include environmental persistence produced results that were highly similar to those of the primary model even with wide-ranging values that were included in the sensitivity analysis (bootstrapped Pearson’s correlation, r = 1.00, 0.97, 0.68 corresponding to a national and annual average proportion of 2.2 × 10^−62^ of viruses surviving after 100 days based on prevailing literature, and values of 6.8 × 10^−7^, and 0.24 of viruses surviving after 100 days due to uncertainty in this metric (see Appendix S1 for methods). Given similar results of the two versions of the model and the need for significant additional data on the amount of virus that can survive in the environment under different conditions and durations of time, we chose to present the more parsimonious version in the main text and place the adapted model in Supplemental Appendix S1.

### Validation

**Figure 5:**
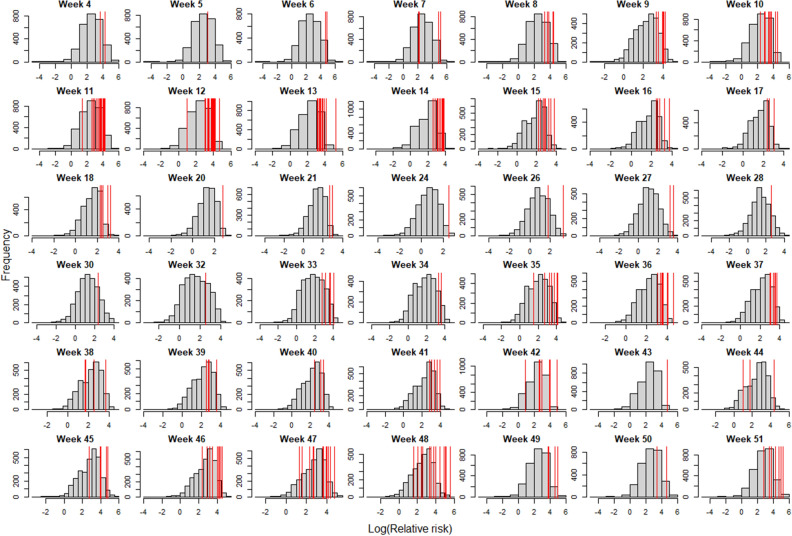
Comparison of the relative risk of counties that experienced spillover events to all counties. Grey bars show the distribution of relative risk scores, log-base 10 transformed, for all counties, while red lines show the relative risk for counties that experienced spillover events. Most spillover counties had risk scores greater than the average of all counties and very few had below-average risk.

The model performed quite well at predicting spillover events during the 2022–2023 HPAI outbreak at the county level (Fig. 4), even though it was not designed to predict spillover events for this novel incursion. Both the AUC and Spearman’s correlation validation methods performed well (Table 1), and outbreaks overwhelmingly occurred in counties with greater than average risk (Fig. 5). This was not true of the fine-scale model where many of the grid cells that actually did experience spillover events were incorrectly assigned no risk by the model because they did not contain poultry operations via the poultry hybrid model. As a reminder, this poultry model simulates locations of likely poultry locations, particularly for the backyard flocks.

**Table 1:** Model validation metrics, area under the receiver operator curve (AUC) and Spearman’s correlation coefficient (Rho) for (A) the full model (county-level spatial scale). In addition to the full model on all poultry farms, the model and validation steps were also run with only (B) commercial poultry operations, for which we have more confidence in the actual locations, and (C) backyard poultry operations for which we have less confidence in spatial locations.

Model sets and model components for validation	AUC	Rho
(A) All Poultry		
Full Model	0.84	0.58
Number of infected waterfowl	0.81	0.54
Waterfowl abundance	0.82	0.56
Poultry relative risk	0.63	0.21
Count of poultry facilities	0.51	0.01
(B) Commercial Only		
Full Model	0.85	0.60
Number of infected waterfowl	0.84	0.58
Waterfowl abundance	0.84	0.58
Poultry relative risk	0.88	0.65
Count of poultry facilities	0.57	0.11
(C) Backyard Only		
Full Model	0.81	0.52
Number of infected waterfowl	0.77	0.46
Waterfowl abundance	0.76	0.44
Poultry relative risk	0.68	0.30
Count of poultry facilities	0.66	0.28

Certain components of the model were more informative than others (Table 1). Specifically, a model containing only the effect of waterfowl abundance would have performed nearly as well as the full model. Adding the prevalence component did not appear to have improved the model’s predictive ability. The majority of spillover events occurred in spring (late March through mid-May) when species and counties were predicted to have similar, low levels of AIV infections^27^, presenting little variation in this variable for the model to use. If more spillover events had occurred during mid to late autumn when there are large differences in AIV prevalence among waterfowl, the prevalence component may have had a larger effect on driving model results. Moreover, the gsGD H5 virus is antigenically distinct from other H5 viruses and affects a wide range of species, especially compared to typical endemic viruses which often favor specific species groups and circulate along predictable migratory routes. However, virus prevalence may still be useful in predicting the spillover of the LPAI viruses. Examination of model components also showed that the relative risk and number of different types of poultry operations contributed less predictive power compared to waterfowl abundance (Table 1). However, in additional model and validation runs where we explored using only commercial or backyard poultry operations, we found that the poultry inputs were more informative using the commercial poultry inputs, where point locations are better identified at the higher spatial resolutions (3 km vs county level).

**Figure 6:**
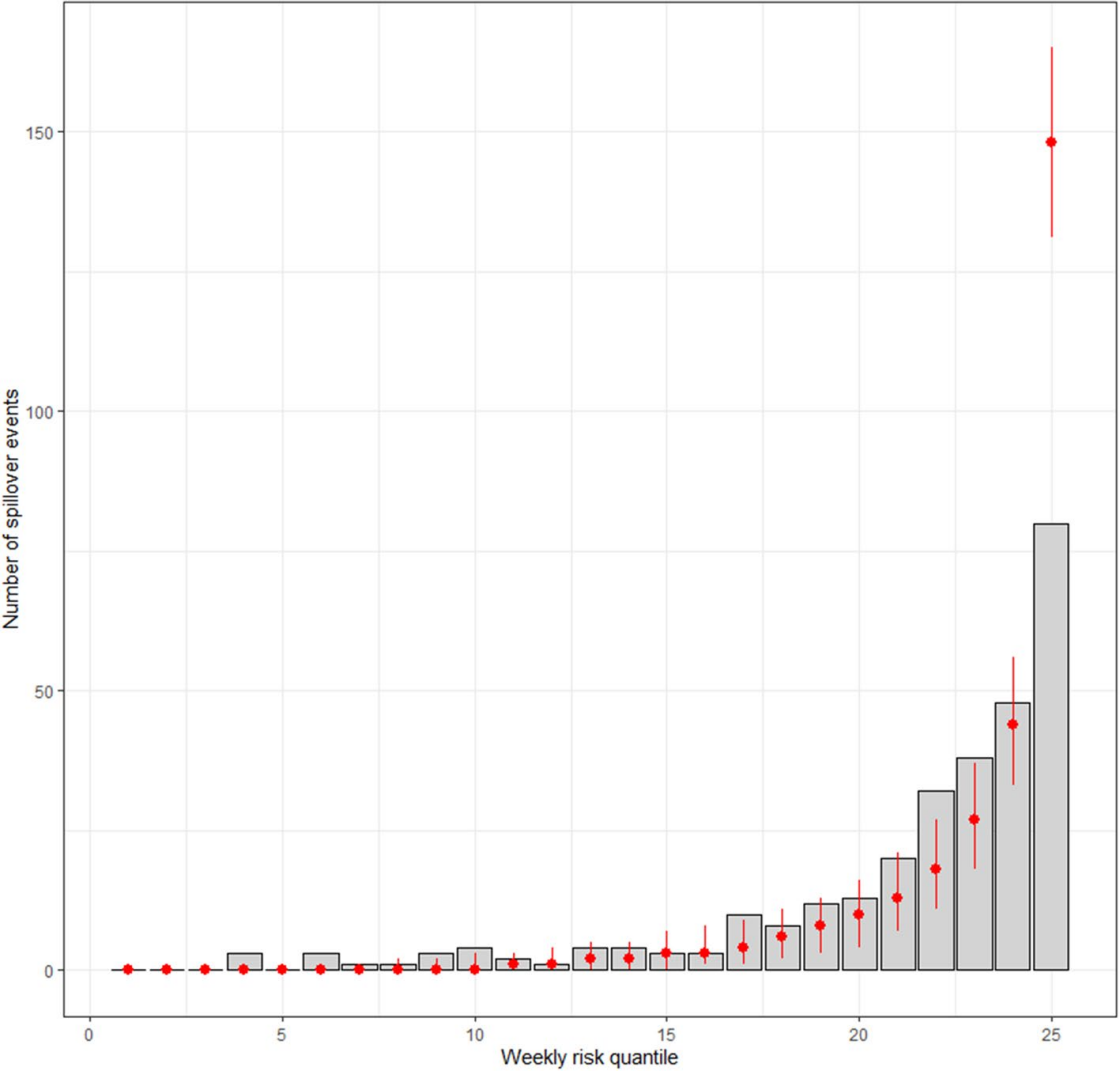
Comparison of model expectations (red bars) to the number of counties that experienced wild bird spillover events in the ongoing highly pathogenic avian influenza H5N1 Clade 2.3.4.4b outbreaks in the United States. Grey bars show the number of counties that experienced a spillover event in each weekly risk quantile. Red points show the average number (± 95% CI) predicted from the model based on simulations. The model performs well for most of the distribution but overestimates transmission risk for the highest quantile risk category.

Lastly, we compared these results to those expected by the model based on simulated outbreak events. The simulations showed similar patterns to the validation data (Fig. 6). However, the simulations tended to place more spillover events into higher-risk categories and fewer spillover events into counties with lower-risk categories than we observed in the actual data. This indicates that the model was likely overconfident in its predictions, particularly at the highest risk quantiles.

## Discussion

In this study we generated predictions of AIV spillover from wild birds to domestic poultry operations at weekly intervals for the contiguous United States, elucidating patterns in the spatio-temporal distribution of transmission risk at the wild-domestic interface. The estimated risk at a given grid cell and time step was primarily driven by waterfowl abundance. Specifically, we observed increases in predicted risk in the spring as waterfowl began the northward migration, followed by declines through the summer breeding period when bird movements are reduced, and a resurgence during fall migration. These trends were further amplified by AIV prevalence, which generally follows the same seasonal patterns^27^ driven primarily by availability of naïve juveniles^28,29^, with contributions from factors such as environmental persistence along migratory routes and wintering sites^30^. Though important, these patterns were not surprising given that previous efforts have indicated the importance of waterfowl abundance to the transmission of AIV across this interface^15,16^.

While the seasonal trends in transmission risk driven by waterfowl abundance and AIV prevalence appear reasonable for the endemic LPAI viruses on which this model was built, we might not expect such patterns during a novel incursion such as the current gsGD H5 outbreak in North America. During a novel incursion, waterfowl will lack homosubtypic immunities that would help mediate viral spread among adults^31^, allowing rapid spread even during periods that would otherwise have low prevalence^32^. This dynamic is well illustrated by the large number of HPAI detections and mortality events in wild birds observed during the summer of 2022 following the initial introduction into the United States in January 2022^6^. Similarly, while endemic LPAI’s do move longitudinally between flyways, spread is predominately latitudinal within a flyway and linked to annual migratory cycles^33,34^. However, during the current gsGD H5 outbreak we have observed an east to west spread based around the initial introduction location in the Atlantic flyway^35,36^. Due to the differing dynamics between endemic viruses and novel incursions, we constrained our validation to only make comparisons spatially within weeks and not between weeks. This same limitation should be considered when comparing predicted risk hotspots across time, as the relative risk between locations within weeks is more informative than the absolute values at a location across time based on the nature of the novel incursion.

Given the strong influence of waterfowl distribution and AIV prevalence on transmission risk across the landscape (Table 1), the impact of domestic poultry distribution appears subdued in comparison (Table 1). This reduced role in the pattern of risk is likely due to the static nature of farm locations which present a spatially consistent interface. Additionally, at first glance, poultry distribution appears to have little variation across the landscape, however, when poultry are broken down by production category (commercial vs. backyard) and commodity type (broiler, turkey, etc.) notable trends emerge. Commercial broiler production appears clustered, with hotspots in regions such as the southeast and the Delmarva Peninsula (Delaware, Maryland, Virginia eastern shore). Meanwhile, turkey production is mainly located in the upper Midwest. Due to the concentrations of poultry production facilities, these areas have elevated transmission risk across the year relative to surrounding regions. Conversely, backyard poultry operations are scattered much more randomly and evenly around the landscape, likely reducing centralization of risk.

An important limitation of the high spatial resolution model sets (3 km^2^) comes from the application of the hybrid poultry model inputs^37^ which are the most recent spatially explicit, high-resolution predictions of poultry operation locations available for the United States. This model provides likely coordinates of poultry farm locations by farm type (broiler, layer, pullet, or turkey; commercial or backyard; and breeding or production) based on environmental covariates, artificial intelligence to identify commercial facilities, and constraints by the number and size of operations reported in the USDA-NASS CoA. The model provides predicted locations, not actual locations, particularly for the smaller farm types (non-commercial and backyard flocks) or those that are located in counties that did not receive the artificial intelligence and satellite imagery application. Hence, at the 3 km scale for our risk models, the poultry layer might show farm locations where they are likely to be, but in reality, are located somewhere in the general vicinity within or just beyond 3 km. When validating with spillover events that are derived from virus isolates collected from actual farm locations, this spatial incongruity shows weaker model performance. However, when collated at a slightly coarser scale, such as the county level, the model performs quite well. Nearly all outbreaks occurred in counties that were identified as being above average risk (Fig. 5), and the AUC and Spearman's correlations show strong predictive power at this spatial scale (Table 1). Still, the model does appear overconfident in counties it deems to be of the highest risk quantile (25th weekly risk quantile, Fig. 6). This overconfidence could be explained by three primary scenarios. First, our model assumes inputs have a linear impact on the effect of risk—such that a doubling of any variable doubles the risk. However, in practice, it is likely that at very large values, a level of risk saturation is reached. Framed another way, if a large number of waterfowl are already present, additional waterfowl likely add negligible additional risk. The second possible explanation for model overconfidence is that the model could simply be overfit due to poultry location data being modeled as exact locations without error. A possible future improvement to the model could be to add error estimates to the poultry model inputs so they could be incorporated stochastically into the risk models. Finally, it is possible that farms in counties with the highest poultry production may be more likely to practice enhanced biosecurity due to the elevated perceived risk that may come from having so many additional farms within a close proximity.

A second major element of our models that could be improved with additional data is refinement of the relative risk scaler used to represent differential risk between poultry farms based upon the commodity type, sector, and category. Previous work in Europe indicates that backyard farms are at greater risk of an initial introduction event than meat production farms, which are in turn at greater risk than breeder facilities with trends primarily driven by the access of birds in each group to the outdoors^38,39^. Similarly, challenge studies indicate that turkeys are more susceptible to infection than are chickens^40–42^. While these general trends are well supported (see full description in Supplemental Appendix S2), the selection of relative risk scalers used in this study was a rough approximation of true risk based on studies from different countries due to data availability. Although the virus challenge studies from the literature suggest that these patterns are applicable more broadly than what is reported for the Europe poultry risk studies, this is still a notable limitation of the data within our model. Therefore, further investigation of outbreak patterns within the United States could meaningfully improve input values for this portion of our model and thereby improve model estimates. Additionally, it should be noted that our model does not account for presence or abundance of potential mechanisms for introducing virus shed by waterfowl into the actual poultry facilities, such as bridge species^43,44^, indirect or fomite transmission, airborne transmission^45^, etc. These introduction mechanisms were omitted due to insufficient data on the topics.

The modular nature of our underlying model and the demonstrated prediction power, including novel solutions, makes it an effective adaptive tool for the management of AIVs. That is, any component of the model can be further refined as more information becomes available or to account for changes on the ground. For instance, the model as presented here focuses on waterfowl, as they are the primary reservoir of viruses that ultimately spill into poultry facilities. However, as more details become available on influenza prevalence, particularly with the continuation of the gsGD H5 clade 2.3.4.4b outbreaks in the United States, additional wild taxa might be added. Likewise, additional data on wild bird distributions are increasingly becoming available for a wider variety of taxa, at finer spatial scales, and with adjustments for global warming. These updated estimates of relative waterfowl abundance can easily replace the current data in this model. Even more importantly, though our model performed well at predicting outbreaks of a novel HPAI incursion, something it was not designed to do, additional modifications could be made in the adaptive management of such novel incursions. For instance, if one were to generate hypothetical prevalence models in wild birds representing a novel incursion, such models could replace the prevalence data in the model presented here to simulate incursion events under different scenarios. On the poultry side of the equation, although some actual poultry population data exist for specific geographic areas or poultry sectors, a national, comprehensive database of U.S. poultry operations is lacking. The hybrid model used here^37^, and FLAPS^46^ provide population datasets of farm location and type suitable for modeling in the absence of true farm location data. Any updates to those datasets (e.g. updated USDA-NASS CoA), or datasets collected and made available even at a state or regional level, could be incorporated into the interface models to improve spatial accuracy of poultry farms. However, this could be substituted out for other publicly available data^47^, or in the event that a poultry producer or contract growing operation wished to analyze their own risk, could be replaced with their own proprietary data of exact farm locations and the biosecurity measures taken at each. Finally, new components could easily be added to this model to extend it in a multitude of ways, as we demonstrate with environmental persistence of the H5 and H7 subtypes.

The models presented here provide detailed identification of the spatio-temporal transmission risk of avian influenza from wild waterfowl to domestic poultry for the contiguous United States. Our results highlight that transmission risk is driven primarily by wild waterfowl abundance and AIV prevalence, but that poultry distribution, farm type, and the poultry species being raised are all important considerations. While there are clear areas for improvement to the model inputs as identified above, the strong performance of these models, as validated by the ongoing gsGD H5 outbreak, demonstrates their utility for managers and response coordinators who may seek to use these models to help stage response resources or inform enhanced surveillance approaches. Providing the best science possible in emergency response situations and preferably in anticipation of future emergence of novel pathogens may be key to protecting human, agriculture, and wildlife health. In order to do so, an adaptive approach needs to be taken^5^, whereby learning from past events and endemically circulating non-pathogenic viral forms, in combination with leveraging early but scarce outbreak data is an important first step in providing tools to managers and decision makers^9^. Next steps for providing adaptive models for the rapidly evolving gsGD H5 clade 2.3.4.4b situation in North and South America^48,49^, would be to (a) improve the existing baseline data distribution inputs for host species and farm types, (b) update spatial and temporal inputs reflecting evolving virus patterns across the diverse range of infected taxa, (c) provide risk rankings across poultry types based on data from this region, and (d) extend models across political borders for a cross-continental resource (Canada through South America).

## Materials and methods

### Modeling framework

Here we aim to quantify variation in both the spatial and temporal risk of an avian influenza virus spillover event from wild reservoir species into domestic poultry operations. As such spillover events are generally not reported in the United States for viruses of low-pathogenicity and incursions of highly pathogenic viruses have historically been rare, there is a dearth of data to generate such predictions using conventional regression-type models. As such, our deterministic approach is based on epidemiological theory, following similar past work in other regions^23,50^.

The risk of such a spillover event can be modeled as the relative transmission risk (*RR*) between wild birds and domestic poultry operations using the following equations:

1 $$RR=W\times P$$

where *W* is the estimated number of infected waterfowl present at a given time and place and *P* is the number of poultry operations in that location weighted by their risk of a spillover event occurring. This equation can be further broken down to:

2 $$RR=\sum_{s}\left({Wa}_{s}\times {Wp}_{s}\right)\times \sum_{t}\left({Pn}_{t}\times {Pr}_{t}\right)$$

where *Wa*_*s*_ is the relative abundance of waterfowl of species *s*, *Wp*_*s*_ is the percentage of those waterfowl predicted to be positive with AIVs, *Pn*_*t*_ is the number of poultry operations of a given type *t*, and *Pr*_*t*_ is the relative risk associated with each operation type (See Fig. 1 for graphical depiction of model components). Each of these component variables is described in detail below. In addition to these components, we also developed a set of models that included environmental persistence. However, these models are based on weaker assumptions about the nature of environmental persistence, and as we found little impact of this component on the overall model, even when including sensitivity measures for varying the amount of environmental persistence, these alternative models have been placed in Supplemental Appendix S1.

### Model components

Original seasonal estimates of waterfowl abundance (*Wa*_*s*_) were developed for 10 common dabbling duck species in the genus *Anas*^24^ using Bayesian joint spatio-temporal hierarchical models and eBird citizen science data ^51^. More recently, predicted relative abundance models have become available via eBird Status & Trends [https://ebird.org/science/status-and-trends;^25^] and we switched to these weekly spatial layers as one of our model inputs. In short, these models provide estimated relative abundance of each species (the expected count of individuals detected by an expert birder on a 1 h, 1 km transect at an optimal time of day, which is likely lower than true abundance due to detection issues). While these values may not be exact counts of individuals present, they provide a strong “relative” index by which we can compare waterfowl abundance across the landscape. These predictions are estimated for all 52 weeks of the year at a spatial resolution of 2.96 km^2^. In total, we included estimates for 30 common waterfowl (Anatidae) species, which included all species for which we have spatial and temporal prevalence estimates (below). Comparing relative abundance among multiple species can be problematic, as these methods do not account for species-specific differences in detection probability. However, this should be mitigated here as most waterfowl species are of roughly comparable size, visibility, and behavior though there are some inherent differences between species.

The estimated percentage of waterfowl infected with AIV (*Wp*_*s*_), by species, was developed at weekly intervals in a previous model of AIV prevalence in wild waterfowl^27^. In short, that model combined data from the National Wild Bird Surveillance Program^52,53^ and the Influenza Research Database^54,55^ for 30 species of waterfowl. The model was then used to generate spatiotemporal predictions of the proportion of birds that would test positive for AIVs (low pathogenicity) for 30 waterfowl species at weekly intervals for each county in the United States.

The number and type of poultry operations (*Pn*_*t*_) comes from the “hybrid poultry model” developed by the U.S. Department of Agriculture (USDA)^37^. This model uses a baseline population from the Farm Location and Agricultural Production Simulator (FLAPS) that combines a poultry farm suitability surface with poultry farm numbers and types from the USDA, National Agricultural Statistics Service (NASS), Census of Agriculture (CoA), to stochastically place likely farm locations and assign them an operation type^46^. Operation type included the commodity type (broiler, layer, pullet, or turkey), sector (breeding or production), and category (backyard or commercial, numbering 271,289 and 42,019, respectively). In 594 U.S. counties with the greatest poultry production, the hybrid model incorporated remote sensing and artificial intelligence to detect buildings likely to be commercial poultry farms to improve the accuracy of the FLAPS predictions. Though there are likely some changes in poultry production numbers throughout the year (e.g., downtime between flock placement), these are static spatial data and have no temporal component. Since this source data only includes turkey and chicken farms by type, other farm types such as game birds and ducks are not individually represented in this effort.

Finally, as there is known variation in the relative risk of different farm types, we developed a relative risk scaler for each poultry operation type. These are described in detail in Supplemental Appendix S2. In short, this includes knowledge on risk by species and poultry type gained from several European studies (where data are available), as well as differences in risk between birds being housed indoors and outdoors. Note that our model does not account for presence or abundance of potential mechanisms for introducing virus shed by waterfowl into the actual poultry facilities due to lack of available data.

It has been well documented that different avian influenza virus strains vary with regard to differential risks, even within subtype. Of particular note are the H5 and H7 subtypes (defined by the hemagglutinin surface protein) which have the potential to mutate and become highly pathogenic after spilling over into domestic poultry^56^. As such, we developed an additional pair of models focused on these two agriculturally important AIV subtypes. For these models, the overall risk was multiplied by the proportion of viruses expected for each subtype based on data from Kent et al.^57^. This study presented the proportion of influenza positive birds expected to be infected with either the H5 or H7 subtype (both highly and low pathogenic strains) at the county level at monthly intervals for seven waterfowl species. As the taxonomic breadth of the available study was lower than that of our current efforts and because limited species differences were detected, for model input we averaged risks across all species. Additionally, the Kent et al.^57^ study provided predictions at monthly, instead of weekly intervals, so we interpolated these predictions to place them on the same weekly temporal scale as our models.

In summary, four model sets were developed in this study: (a) primary models that are driven by LPAI endemically circulating waterfowl prevalence, (b) models targeted for H5 subtypes, (c) models targeted for H7 subtypes, and (d) the primary model set with environmental persistence components added to the equation. Each model set was conducted at two spatial scales: (a) ~ 2.96 km^2^ based on the waterfowl inputs (hereafter, 3 km^2^), and (b) county level which aligns with the waterfowl prevalence models^27,57^, the USDA hybrid poultry model^37^ calibrations, and the scale to which USDA targets AIV management and response. While an intermediate scale such as zip code^58^ was considered, these models were excluded to simplify results given mismatch in scale for poultry predictions and validation data (see below).

### External validation

These models are designed to estimate the risk of spillover of endemic AIVs, generally of low-pathogenicity, from wild birds into domestic poultry operations, based on the spatio-temporal AIV prevalence model inputs^27,57^. Data necessary to validate a model such as this require distinction between wild bird farm introductions versus farm-to-farm transmission (lateral spread) or common source events and are generally not available or accessible in most regions of the globe, let alone the United States. However, the 2022 incursion of a gsGD H5N1 clade 2.3.4.4b into the United States^49,59^ provided us with viral sequences from infected premises which were used in phylogenetic analyses to identify wild bird spillover events to validate our model in an HPAI context.

In collaboration with USDA, results from phylogenetic analyses that identified evidence for wild bird introduction events into poultry flocks provided censored location-specific inputs for our model validation^49^. To differentiate between initial wild bird introductions (spillovers) and lateral (farm to farm transmissions) or common source transmission, researchers from the USDA and their partners analyzed samples collected from wild birds and domestic poultry and screened via real-time reverse transcription polymerase chain reaction (RT-PCR) targeting conserved regions of the matrix gene. Non-negative samples were then confirmed and sequenced by the USDA National Veterinary Services Laboratories. These data, along with other known subtypes from previous outbreak events were then included in a Basic Local Alignment Search Tool (BLAST) analysis within the Global Initiative for Sharing All Influenza Database (GISAID). All samples were classified by genotype utilizing Maximum Likelihood phylogenetic tree generation or the BLAST based GenoFLU tool^49^. Bayesian time-scaled phylogenetic analysis produced final differentiation between potential farm-to-farm transmission events versus independent introductions from wild birds. Full details on methodology can be found in Youk et al.^49^. The final validation dataset represented 294 spillover events from 176 counties. It should be noted that while this dataset provides a confirmation date for infection, we manually set the date of assumed viral introduction as 14 days prior to confirmation to allow for the flock-level incubation period and delay to confirmation^60^. We acknowledge that various factors such as level of surveillance and strain specific pathogenicity could impact the accuracy of this value but feel that the possible biases introduced would likely have minimal impact on interpretation.

This validation dataset is not equivalent to the interface transmission risk model predictions (wild bird spillover introductions into poultry operations using inputs based on endemically circulating AIVs^27,57^ given availability of data), so some limitations should be noted. First, the patterns of endemically circulating LPAI in reservoir host species may show different seasonal patterns than those from a novel incursion. While past literature shows North American H5 prevalence peaking in the autumn^27,28,52^, the gsGD virus, which is antigenically distinct from North American H5 viruses, continued to rise throughout the spring and summer. Only a small decrease was noted during the summer months, followed by another peak that arose in the fall 2022^6,49^. As such, we did not validate this model based on changes at a given location or in overall risk across calendar weeks of the year. Instead, we constrained our validation steps to examine patterns within a given weekly interval until spatiotemporal prevalence models for this novel incursion can be developed. The spatial pattern of this incursion began with introduction along the East Coast of North America with subsequent spread south and westward across the continent to the Pacific Coast^36,59^; however, several virus introductions were characterized from both the Atlantic and Pacific flyways and the virus expansion, and emergence of reassortments has not been strictly linear^35,49^. Due to its spatial and temporal complexity, we did not attempt to incorporate the gsGD H5 expansion (spread from east to west) in our validation process. This conservative decision should result in low validation estimates, meaning that the predictive power of the model was likely stronger than what is identified by the validation exercise.

Model validation took place at two spatial scales. First, we obtained our specific risk estimates for each outbreak location at the fine, 3 km^2^, resolution of our full model. We then aggregated all data to the county level to understand how well the model performed at this coarser spatial scale which better aligns with larger response and planning efforts^61^. Inference on model validation was based on two metrics. First, we used the area under the receiver operating characteristic curve (AUC) using the package *PresenceAbsence*^62^ to understand the model’s ability to correctly identify counties that experienced a spillover event. This value was based on whether a county experienced a spillover event in a given week and the associated ranked relative risk for that county during that week. We also used a Spearman’s correlation to further validate the model. As the number of counties that did not experience an outbreak on a particular week greatly exceeded the number of counties that did, we elected to use a bootstrapping procedure to better reflect the model’s predictive performance. In short, we randomly sampled with replacement the model’s risk of an outbreak both for the counties that did and did not experience a spillover in a given week so that the number pulled equaled the number of spillover events. For example, there were 15 spillover events detected in week 14 (early April) in the raw data, so each permutation would contain 30 counties (15 that did and 15 that did not experience a spillover event during week 14). These values were then used to calculate a Spearman’s correlation of the ranked relative risk of a spillover event for a given county in a given week and whether or not it experienced an outbreak. This procedure was run for 1,000 iterations and the values were averaged. This series of validations was performed to allow us to assess predictive ability of our model.

In addition to running these validation steps for the model’s overall relative risk, we also ran them for individual components of the model to quantify their contribution to its predictive performance. This included the number of infected waterfowl ($W$) in Eq. 1, the relative abundance of waterfowl (the sum of ${Wa}_{s}$ in Eq. 2), the number and relative risk of poultry operations (${Pr}_{t}$ in Eq. 1), and the raw number of poultry operations (the sum of ${Pn}_{t}$ in Eq. 2).

Finally, we also wanted to examine how well the model performed compared to its own expectations, which we accomplished using a simulation procedure and qualitative, graphical comparison^63^. For each week of the year for which we had validation data, we randomly selected counties, with replacement, to experience a “spillover event” equal to the number of spillover events in that week, with the probability of a county being selected proportional to its predicted relative risk. This procedure was run for 1,000 iterations. We then compared the number of outbreaks that occurred in counties of different risk quantiles compared to that in the simulated data. This series of validations was performed to allow us to assess model confidence versus performance (i.e., the expected versus observed distribution of risk values at spillover locations).

### Supplementary Information

Supplementary Information 1.

Supplementary Information 2.

## Data Availability

All data supporting this manuscript as well as all final model outputs are freely available^26^. The only exception to this is specific outbreak locations used for model validation which are not released publicly to protect privacy of individual farmers.

## References

[1] Stallknecht DE (2003). Ecology and epidemiology of avian influenza viruses in wild bird populations: waterfowl, shorebirds, pelicans, cormorants, etc. Avian Dis..

[2] Sonnberg S, Webby RJ, Webster RG (2013). Natural history of highly pathogenic avian influenza H5N1. Virus Res..

[3] Pohlmann A (2022). Has epizootic become enzootic? Evidence for a fundamental change in the infection dynamics of highly pathogenic avian influenza in Europe, 2021. mBio.

[4] Verhagen JH, Fouchier RAM, Lewis N (2021). Highly pathogenic avian influenza viruses at the wild-domestic bird interface in Europe: Future directions for research and surveillance. Viruses.

[5] Ramey AM (2022). Highly pathogenic avian influenza is an emerging disease threat to wild birds in North America. J. Wildl. Manag..

[6] USDA. Detections of highly pathogenic avian influenza in wild birds. https://www.aphis.usda.gov/aphis/ourfocus/animalhealth/animal-disease-information/avian/avian-influenza/hpai-2022/2022-hpai-wild-birds (2023).

[7] USGS. Wildlife health information sharing partnership event reporting system. https://whispers.usgs.gov/home (2023).

[8] USDA. Confirmations of highly pathogenic avian influenza in commercial and backyard flocks. https://www.aphis.usda.gov/aphis/ourfocus/animalhealth/animal-disease-information/avian/avian-influenza/hpai-2022/2022-hpai-commercial-backyard-flocks (2023).

[9] Harvey JA, Mullinax JM, Runge MC, Prosser DJ (2023). The changing dynamics of highly pathogenic avian influenza H5N1: Next steps for management & science in North America. Biol. Conserv..

[10] Boender GJ (2007). Risk maps for the spread of highly pathogenic avian influenza in poultry. PLoS Comput. Biol..

[11] Scott AB (2018). Low- and high-pathogenic avian influenza H5 and H7 spread risk assessment within and between Australian commercial chicken farms. Front. Vet. Sci..

[12] Andronico A (2019). Highly pathogenic avian influenza H5N8 in south-west France 2016–2017: A modeling study of control strategies. Epidemics.

[13] La Sala LF (2019). Spatial modelling for low pathogenicity avian influenza virus at the interface of wild birds and backyard poultry. Transbound. Emerg. Dis..

[14] Mulatti P (2018). Integration of genetic and epidemiological data to infer H5N8 HPAI virus transmission dynamics during the 2016–2017 epidemic in Italy. Sci. Rep..

[15] Humphreys JM (2020). Waterfowl occurrence and residence time as indicators of H5 and H7 avian influenza in North American Poultry. Sci. Rep..

[16] Gierak A, Śmietanka K (2021). The impact of selected risk factors on the occurrence of highly pathogenic avian influenza in commercial poultry flocks in Poland. J. Vet. Res..

[17] Martin GR (2011). Understanding bird collisions with man-made objects: A sensory ecology approach. Ibis.

[18] Kane MJ, Price N, Scotch M, Rabinowitz P (2014). Comparison of ARIMA and random forest time series models for prediction of avian influenza H5N1 outbreaks. BMC Bioinform..

[19] Barnes B, Glass K (2018). Modelling low pathogenic avian influenza introduction into the commercial poultry industry. Math. Biosci..

[20] Belkhiria J, Alkhamis MA, Martínez-López B (2016). Application of species distribution modeling for avian influenza surveillance in the United States considering the North America migratory flyways. Sci. Rep..

[21] Belkhiria J, Hijmans RJ, Boyce W, Crossley BM, Martínez-López B (2018). Identification of high risk areas for avian influenza outbreaks in California using disease distribution models. PLoS ONE.

[22] Yousefinaghani S, Dara RA, Poljak Z, Sharif S (2020). A decision support framework for prediction of avian influenza. Sci. Rep..

[23] Prosser DJ (2013). Mapping avian influenza transmission risk at the interface of domestic poultry and wild birds. Front. Public Health.

[24] Humphreys JM, Murrow JL, Sullivan JD, Prosser DJ (2019). Seasonal occurrence and abundance of dabbling ducks across the continental United States: Joint spatio-temporal modelling for the Genus *Anas*. Divers. Distrib..

[25] Fink D (2020). Modeling avian full annual cycle distribution and population trends with citizen science data. Ecol. Appl..

[26] Prosser, D. J. *et al.* Estimates of avian influenza transmission risk across the wild waterfowl - domestic poultry interface. *U.S. Geological Survey data release, *10.5066/P91AP4XL (2024).

[27] Kent CM (2022). Spatiotemporal changes in influenza A virus prevalence among wild waterfowl inhabiting the continental United States throughout the annual cycle. Sci. Rep..

[28] Hénaux V, Samuel MD, Bunck CM (2010). Model-based evaluation of highly and low pathogenic avian influenza dynamics in wild birds. PLoS One.

[29] Gorsich EE (2021). Continental-scale dynamics of avian influenza in U.S. waterfowl are driven by demography, migration, and temperature. Ecol. Appl..

[30] Breban R, Drake JM, Stallknecht DE, Rohani P (2009). The role of environmental transmission in recurrent avian influenza epidemics. PLoS Comput. Biol..

[31] Fereidouni SR (2009). Highly pathogenic avian influenza virus infection of mallards with homo- and heterosubtypic immunity induced by low pathogenic avian influenza viruses. PLoS One.

[32] Antia R, Halloran ME (2021). Transition to endemicity: Understanding COVID-19. Immunity.

[33] Lam TTY (2012). Migratory flyway and geographical distance are barriers to the gene flow of influenza virus among North American birds. Ecol. Lett..

[34] Fourment M, Darling AE, Holmes EC (2017). The impact of migratory flyways on the spread of avian influenza virus in North America. BMC Evol. Biol..

[35] Alkie TN (2022). A threat from both sides: Multiple introductions of genetically distinct H5 HPAI viruses into Canada via both East Asia-Australasia/Pacific and Atlantic flyways. Virus Evol..

[36] Bevins S (2022). Intercontinental movement of highly pathogenic avian influenza A(H5N1) clade 2.3.4.4 virus to the United States, 2021. Emerg. Infect. Dis..

[37] Patyk KA (2020). Modelling the domestic poultry population in the United States: A novel approach leveraging remote sensing and synthetic data methods. Geospat. Health.

[38] Bouwstra R (2017). Risk for low pathogenicity avian influenza virus on poultry farms, the Netherlands, 2007–2013. Emerg. Infect. Dis..

[39] Gonzales JL (2010). Low-pathogenic notifiable avian influenza serosurveillance and the risk of infection in poultry—A critical review of the European Union active surveillance programme (2005–2007). Influenza Respir. Viruses.

[40] Mutinelli F, Capua I, Terregino C, Cattoli G (2003). Clinical, gross, and microscopic findings in different avian species naturally infected during the H7N1 low- and high-pathogenicity avian influenza epidemics in Italy during 1999 and 2000. Avian Dis..

[41] Aldous EW (2010). Infection dynamics of highly pathogenic avian influenza and virulent avian paramyxovirus type 1 viruses in chickens, turkeys and ducks. Avian Pathol..

[42] Spackman E (2010). The pathogenesis of low pathogenicity H7 avian influenza viruses in chickens, ducks and turkeys. Virol. J..

[43] Velkers FC, Blokhuis SJ, Veldhuis Kroeze EJB, Burt SA (2017). The role of rodents in avian influenza outbreaks in poultry farms: A review. Vet. Q..

[44] Shriner SA, Root JJ (2020). A review of avian influenza A virus associations in synanthropic birds. Viruses.

[45] Zhao Y (2019). Airborne transmission may have played a role in the spread of 2015 highly pathogenic avian influenza outbreaks in the United States. Sci. Rep..

[46] Burdett CL, Kraus BR, Garza SJ, Miller RS, Bjork KE (2015). Simulating the distribution of individual livestock farms and their populations in the United States: An example using domestic swine (*Sus scrofa domesticus*) farms. PLoS One.

[47] Robinson TP (2014). Mapping the global distribution of livestock. PLoS One.

[48] Kandeil A (2023). Rapid evolution of A(H5N1) influenza viruses after intercontinental spread to North America. Nat. Commun..

[49] Youk S (2023). H5N1 highly pathogenic avian influenza clade 2.3.4.4b in wild and domestic birds: Introductions into the United States and reassortments, December 2021–April 2022. Virology.

[50] Hill A (2019). Quantifying the spatial risk of Avian Influenza introduction into British poultry by wild birds. Sci. Rep..

[51] Sullivan BL (2009). eBird: A citizen-based bird observation network in the biological sciences. Biol. Conserv..

[52] Bevins SN (2014). Large-scale avian influenza surveillance in wild birds throughout the United States. PLoS One.

[53] Deliberto TJ (2009). Surveillance for highly pathogenic avian influenza in wild birds in the USA. Integr. Zool..

[54] Squires RB (2012). Influenza Research Database: An integrated bioinformatics resource for influenza research and surveillance. Influenza Respir. Viruses.

[55] Zhang Y (2017). Influenza Research Database: An integrated bioinformatics resource for influenza virus research. Nucleic Acids Res..

[56] Alexander DJ (2007). An overview of the epidemiology of avian influenza. Vaccine.

[57] Kent CM, Bevins SN, Mullinax JM, Sullivan JD, Prosser DJ (2023). Waterfowl show spatiotemporal trends in influenza A H5 and H7 infections but limited taxonomic variation. Ecol. Appl..

[58] Rorres C, Pelletier STK, Smith G (2011). Stochastic modeling of animal epidemics using data collected over three different spatial scales. Epidemics.

[59] Caliendo V (2022). Transatlantic spread of highly pathogenic avian influenza H5N1 by wild birds from Europe to North America in 2021. Sci. Rep..

[60] World Organization for Animal Health. *Terrestrial Animal Health Code*. (2023).

[61] USDA. Highly Pathogenic Avian Influenza (HPAI). https://www.aphis.usda.gov/aphis/ourfocus/animalhealth/emergency-management/hpai/fadprep-hpai (2023).

[62] Freeman EA, Moisen G (2008). PresenceAbsence: An R Package for presence absence analysis. J. Stat. Softw..

[63] Murray-Smith DJ (1998). Methods for the external validation of continuous system simulation models: a review. Math. Comput. Model. Dyn. Syst..

