# Supplementary material for: Using an adaptive modeling framework to identify avian influenza spillover risk at the wild-domestic interface
Source: Sci Rep. 2024 Jun 20;14:14199. doi: 10.1038/s41598-024-64912-w (PMC11189914; doi:10.1038/s41598-024-64912-w)
Supplement: Supplementary file 1 — Supplementary Information 1. [file 41598_2024_64912_MOESM1_ESM.pdf]

Supplemental Appendix S1

to

Using an adaptive modeling framework to identify avian influenza spillover risk at the wild-domestic interface

Diann J. Prosser[1,*], Cody M. Kent[2,3,4], Jeffery D. Sullivan[1], Kelly A. Patyk[5], Mary-Jane McCool[5], Mia Kim Torchetti[6], Kristina Lantz[6], Jennifer M. Mullinax[3]

[1] U.S. Geological Survey, Eastern Ecological Science Center, Laurel, MD 20708

[2] Volunteer to the U.S. Geological Survey, Eastern Ecological Science Center, Laurel, MD, 20708

[3] Department of Environmental Science and Technology, University of Maryland, College Park, MD 20742

[4] Department of Biology, Frostburg State University, Frostburg, MD 21532

[5] U.S. Department of Agriculture, Animal Plant and Health Inspection Service, Veterinary Services, Strategy and Policy, Center for Epidemiology and Animal Health, Fort Collins, CO 80521

[6] National Veterinary Services Laboratories, Animal and Plant Health Inspection Service, USDA, Ames, IA 50010

*Corresponding author: Diann Prosser; dprosser@usgs.gov

This research was supported by the USDA Animal and Plant Health Inspection Service (Cooperative Agreement 6000001762), the U.S. Geological Survey Ecosystems Mission Area, and the National Science Foundation (NSF) “PIPP Phase 1: International Center for Avian Influenza Pandemic Prediction and Prevention” (no. 2200310). Use of trade, firm, or product names is for descriptive purposes only and does not imply endorsement by the U.S. Government. The findings and conclusions in this publication are those of the authors and should not be construed to represent any official USDA or U.S. Government determination or policy but do represent the views of the U.S. Geological Survey.

Supplemental Appendix S1: Environmental Persistence Models

In addition to the base model, we developed a version of the model to account for environmental persistence. Specifically, this model sought to include the risk of virus remaining active in the substrate, and then at a later date entering a poultry operation and causing a spillover event.

Methods

To model environmental persistence, we modified the general risk equation (Equation 2) to account for viruses that were persisting in the environment, such that the risk at time w (R_w)

$$R_w = R_d + f(R_{w-1})$$

can be modeled as the sum of the risk of direct transmission (R_d; the risk from equation 2) and the risk persisting from the previous week $f(R_{w-1})$, where the risk from the previous week has declined based on a decay function.

There is disagreement in the literature over the extent to which avian influenza viruses are able to persist in the environment. Much of the available literature points towards low persistence, (e.g., 95% of viruses being non-viable after 3 days; Martin et al., 2018), though some studies show viable viruses for extended periods (e.g., after 7 months; Ramey et al., 2020). Here, we base our primary version of this model off the results of a meta-analysis conducted by Martin et al.(2018). Operationally, we implemented the decay function drawn from their meta-analysis with the following equation:

$$f(R_{w-1}) = R_{w-1} \times e^{-1 \times a_w \times k \times 7}$$

where a_w is a matrix of persistence values for each week (w), k is a constant that allows us to increase or decrease the amount of persistence (proportion of viruses that survive from one time step to the next), and 7 is the number of days in between our weekly timesteps. The equation for a_w comes directly from Martin et al.(2018), as

$$a = e^{(\beta_t \times t + \beta_s \times s + \beta_{ts} \times t \times s)}$$

where $\beta_t = 0.023$, and is the effect of temperature (t) on persistence, $\beta_s = -0.019$, and is the impact of salinity (s) on persistence, and $\beta_{ts} = 0.001$ and is the interaction between temperature and salinity.

Temperature data was derived from MODIS 8-day surface temperatures (https://lpdaac.usgs.gov/products/mod11a2v006/). We downloaded data from 2016-2021 and averaged both across years and between daytime highs and nighttime lows. We then selected data from areas that overlap water, to limit the analysis to water surface temperatures, based on the U.S. Geological Survey Gap Analysis Project (https://doi.org/10.5066/F7JM28J1). Salinity data was also taken from the Gap Analysis Project, where we classified "fresh" water as having a salinity of 1 ppt and brackish water assigned a salinity of 25 ppt. Values for both surface temperature and salinity were then interpolated with a moving window average to fill in gaps where water was not present, on the assumption that waterfowl would only occupy areas in and around water.

We then ran the model forward in time for 1,000 weeks and checked if values had stabilized. Additionally, as there is a range in values in the literature on the extent of environmental persistence, we tested the sensitivity of the model to different levels of persistence by altering the value of k (0.1 and 0.01). Weekly results were visually inspected and compared to results from the base model with a Pearson's correlation.

Results

Unsurprisingly, as Martin et al. (2018) expects little and short-lived environmental persistence, predictions of the environmental persistence model did not differ meaningfully from those in the base model without environmental persistence (r = 1.000). As there is some disagreement in the literature as to what extent these viruses are able to persist, we ran additional versions of the model setting *k* to 0.1 and 0.01 respectively to increase environmental persistence. This corresponds to an average proportion of viruses persisting to the next week for each of the three models (*k* = 1, 0.1, 0.01) of 0.00004, 0.37, and 0.91 respectively, or 2.2x10-62, 6.8x10-7, and 0.24, after 100 days. Despite these changes, we still saw only a limited decrease in the correlation between the persistence models and the base model, with Pearson's correlations among all grid cells and weeks among the two models of 1.00, 0.97, and 0.68 respectively. The minor differences appear to be primarily driven by the persistence of some low-level of risk in areas where waterfowl spend some time of the year, but later vacate.

Literature Cited

Martin, G., Becker, D. J., & Plowright, R. K. (2018). Environmental persistence of influenza H5N1 is driven by temperature and salinity: Insights from a Bayesian meta-analysis. In *Frontiers in Ecology and Evolution* (Vol. 6, Issue SEP). Frontiers Media S.A. https://doi.org/10.3389/fevo.2018.00131

Ramey, A. M., Reeves, A. B., Drexler, J. Z., Ackerman, J. T., De La Cruz, S., Lang, A. S., Leyson, C., Link, P., Prosser, D. J., Robertson, G. J., Wight, J., Youk, S., Spackman, E., Pantin-Jackwood, M., Poulson, R. L., & Stallknecht, D. E. (2020). Influenza A viruses remain infectious for more than seven months in northern wetlands of North America. *Proceedings of the Royal Society B: Biological Sciences*, *287*(1934), 20201680. https://doi.org/10.1098/rspb.2020.1680
