# Supplementary material for: Using an adaptive modeling framework to identify avian influenza spillover risk at the wild-domestic interface
Source: Sci Rep. 2024 Jun 20;14:14199. doi: 10.1038/s41598-024-64912-w (PMC11189914; doi:10.1038/s41598-024-64912-w)
Supplement: Supplementary file 2 — Supplementary Information 2. [file 41598_2024_64912_MOESM2_ESM.pdf]

Supplemental Appendix S2

to

Using an adaptive modeling framework to identify avian influenza spillover risk at the wild-domestic interface

Diann J. Prosser[1,*], Cody M. Kent[2,3,4], Jeffery D. Sullivan[1], Kelly A. Patyk[5], Mary-Jane McCool[5], Mia Kim Torchetti[6], Kristina Lantz[6], Jennifer M. Mullinax[3]

[1] U.S. Geological Survey, Eastern Ecological Science Center, Laurel, MD 20708

[2] Volunteer to the U.S. Geological Survey, Eastern Ecological Science Center, Laurel, MD, 20708

[3] Department of Environmental Science and Technology, University of Maryland, College Park, MD 20742

[4] Department of Biology, Frostburg State University, Frostburg, MD 21532

[5] U.S. Department of Agriculture, Animal Plant and Health Inspection Service, Veterinary Services, Strategy and Policy, Center for Epidemiology and Animal Health, Fort Collins, CO 80521

[6] National Veterinary Services Laboratories, Animal and Plant Health Inspection Service, USDA, Ames, IA 50010

*Corresponding author: Diann Prosser; dprosser@usgs.gov

This research was supported by the USDA Animal and Plant Health Inspection Service (Cooperative Agreement 6000001762), the U.S. Geological Survey Ecosystems Mission Area, and the National Science Foundation (NSF) “PIPP Phase 1: International Center for Avian Influenza Pandemic Prediction and Prevention” (no. 2200310). Use of trade, firm, or product names is for descriptive purposes only and does not imply endorsement by the U.S. Government. The findings and conclusions in this publication are those of the authors and should not be construed to represent any official USDA or U.S. Government determination or policy but do represent the views of the U.S. Geological Survey.

Supplemental Appendix S2: Poultry Sector Risk Literature Review

The goal of the following exercise is to provide reasonable estimates for the different levels of risk faced by different types of poultry operations. To generate these estimates we sought to identify risk values from the literature and apply them to U.S. poultry categories. This was selected over a formal meta-analysis as there are only a handful of relevant literature sources, the majority of which rely on the same underlying dataset. Instead, we seek to make reasonable estimates of risk from a more qualitative approach given that stronger estimates are not available for the United States. What follows is a description of the steps taken to reach these estimates, beginning with a review of the current literature, and is followed by a summary of this literature based on European data, transferring these values into the categories used in the United States, and a set of caveats for these estimates.

Literature Overview

Four central publications are relevant to setting values for avian influenza virus (AIV) risk by poultry type. Three (Bouwstra et al., 2017; Gonzales et al., 2010, 2012), are by the same research group, largely re-analyzing the same surveillance data from the European Union/ Netherlands with additional information in each publication based on an updated data set and each taking a different modeling approach. Galletti et al., (2018), does a similar analysis for data collected in Italy. In general, these four studies agree that chickens are less likely to experience initial outbreak events than turkeys (Table 1). In addition, they tend to point to an increase in risk for birds kept outdoors. It should be noted that because of the small sample size, all duck estimates for the four papers have wide confidence intervals. Our model does not include ducks, making this large error irrelevant for the current project.

These values are largely supported by AIV challenge studies (Aldous et al., 2010; Mutinelli et al., 2003; Spackman et al., 2010). General findings are that chickens are far less susceptible than turkeys, but that susceptibility in ducks is highly variable depending on strains. As such, it is reasonable to assume that the trends found for these four European studies are not Europe-specific, but are more broadly applicable. That leaves us with the task of combining these studies in the best way possible.

Table 1. Risk of initial outbreak events by poultry farm type. RR = "relative risk" OR = "odds ratio." Colors range from lowest risk (dark blue) to highest risk (dark red). Missing values represent poultry categories not included in a given paper.

	Farm Type	Risk of initial outbreak			
Species	Flock	Gonzales 2010 (RR)	Gonzales 2012 (RR)	Bouwstra 2017 (RR)	Galletti 2018 (OR)
Chicken	Breeders	1	0.3		
	Breeder (layer)			0.5	
	Breeder (broiler)			0.4	
	Pullets		0.7		
	Layers	1.08			
	Layers (indoors)		1	1	0.72
	Layers (outdoors)		11.1	6.3	
	Broilers	0.25	0	0.2	
Turkey	Turkey	1.64	7.7		1.2
	Meat			12	
	Breeder			11.3	
Mixed	Backyard	0.46			3.4
Duck	Duck/Geese	18.82			1.81
	Meat Duck/indoors		12.8	39.5	
	Breeders/outdoors		24.5	25.5	
Other	Game birds	6.98			0.41
	Ratites	4.8			
	Others	12.8			

The available data to generate risk estimates by poultry type. Hill et al. (2019) generated a poultry risk map for Great Britain. This study uses values from the Gonzales et al., 2010, and 2012 papers (Table 2). Hill et al. (2019) categorizes poultry inputs by species and housing (indoor/outdoor). Although decisions made for indoor birds look reasonable, it should be noted that there are missing data for indoor geese, with their value set to be the same as indoor ducks. Based on AIV surveillance in wild birds, this is likely an overestimate and that they should be at lower risk than ducks. Additionally, the risk of outdoor game birds varies greatly by study, and it is not clear that this estimate is accurate, with limited data comparing the risk of backyard birds to larger operations. However, we do not explicitly include ducks, geese, or game birds in our model. Perhaps the biggest challenge with these estimates is the lack of data for outdoor turkeys. The reasoning behind the value in Hill et al. (2019) is that if indoor turkeys are at greater risk than indoor chickens, then outdoor turkeys must be at least as at risk as outdoor chickens. As such, this is almost certainly a considerable underestimate.

Table 2. Risk values taken from Hill et al., 2019.

Poultry type	Relative Risk	Notes
Indoor chicken	1	Reference category
Indoor turkey	7.7	Direct from Gonzales 2012
Indoor duck	12.8*	Direct from Gonzales 2012
Indoor goose	12.8[a]	Assumed to be the same as duck
Outdoor chicken	11.1	Direct from Gonzales 2012
Outdoor turkey	11.1[b]	Likely an underestimate. Matched with outdoor chickens
Outdoor duck	24.5*	Direct from Gonzales 2012
Outdoor goose	24.5[a]	Assumed to be the same as duck
Outdoor game bird	11.1[c]	Mix of Chickens from Gonzales 2012 and game birds
Backyard birds	??	No estimate given

*. In all studies, ducks have a very large CI.
a. Goose estimates are taken to be the same as duck estimates.
b. No estimate for outdoor turkey. Used estimate for outdoor chicken. Likely an underestimate.
c. This is the value for outdoor chicken, which is similar to that estimated by Gonzales et al., 2010, for game birds.

Overall, this leaves us with two unresolved decisions that apply to the hybrid model estimates. 1) How should we incorporate outdoor turkeys, and 2) how to assess the risk of backyard birds in general.

Poultry Risk Estimates - European categories

We begin with our best estimates for relative risk by poultry type (Table 3) based on the literature reviewed above, followed by a justification for each. Here we provide a relative risk score that we believe is a reasonable estimate of the actual relative risk, a range of scores that could be easily justified, and notes on a few categories, mostly indicating which categories lack direct measures or otherwise strong evidence behind them. A more detailed justification for these numbers follows the table.

Table 3. Risk value for European poultry categories.

Poultry Type	Relative Risk	Range	Notes
Indoor layer/breeder	1	1	Reference category
Indoor broilers	0.2	0-0.25	
Indoor turkey	7.7	1.64-12	
Indoor ducks	12.8	39.5	
Indoor geese	12.8	<12.8	No direct metric given
Outdoor layers/breeders	11.1	6.3-11.1	
Outdoor broilers*	2.2 (11.1)	0-11.1	No direct metric given
Outdoor turkey	18	11.1-24.5	No direct metric given
Outdoor ducks	24.5	24.5-25.5	
Outdoor geese	24.5	<24.5	No direct metric given
Game birds/other	11.1	6.98-?	

*. Commercial and backyard outdoor broilers likely have different risks, see below for details. A value of 2.2 will be used for commercial birds while 11.1 for backyard birds.

Indoor layer/breeder chicken

Indoor layers and breeder chickens are sometimes separated but often combined in studies of risk. Because of this inconsistency, here we are treating them the same. It is reasonable to assume that breeder farms would have stricter biosecurity, so it may be appropriate to lessen their risk. For the main poultry risk studies they are treated as the reference category, so their relative risk is set to 1. We apply the same approach. Pullets are another indoor chicken type not posted above, but one study (Gonzales et al., 2012) found them to be comparable (RR = 0.7). It could be justified to split them out, though we chose not to do so.

Indoor broilers

The Europe/Dutch papers clearly show broilers to be at lower risk than layers/breeders. Gonzales et al., (2012) found no outbreaks that started with indoor broilers – leading to complete separation, while Gonzales et al., (2010) and Bouwstra et al., (2017) found a small handful, giving relative risks of 0.25 and 0.2 respectively. The latter paper has a more robust analysis, so here we used that value of 0.2. All three papers conclude that this is likely due to the short lifespan of broilers, giving them less time to become infected and spread disease, as well as for a LPAI strain to mutate to HPAI prior to the time of slaughter.

Indoor turkey

The three Gonzales papers find turkeys to be more likely to become infected than chickens. Gonzalez et al., (2010) and (2012) did not separate turkeys by production type and found relative risks of 1.64 and 7.7 respectively. Bouwstra et al., (2017), split turkeys into meat and breeder birds, finding little difference between the two, but a greater overall risk level than in the previous papers (12 and 11.3 respectively). We use the value from Gonzales et al., (2012) because it appears to be the more robust analysis, however, one could justify a higher value. Though not statistically significant, Galletti et al., (2018), found a similar trend where turkey farms were at a greater risk than chicken farms. This finding is further supported by the challenge study literature, which universally concludes that turkeys are more likely to become

infected when exposed to AIV than chickens (Aldous et al., 2010; Spackman et al., 2010). Additional literature points to turkeys having more severe infections (Mutinelli et al., 2003) and have found a lower minimal infectious dose for turkeys than chickens or ducks (Pantin-Jackwood et al., 2017; Pillai et al., 2010)..

Indoor ducks

All estimates for ducks have wide confidence intervals because of the small number of farms, and so some caution is warranted here. However, they do regularly show greater risk than chickens or turkeys. Ducks are identified as higher risk in all three European/Dutch papers (Bouwstra et al., 2017; Gonzales et al., 2010, 2012), and Galletti et al., (2018) shows a similar trend in Italy. There is a wide range of possible explanations given, including the frequency at which indoor ducks may still be allowed outside, the storage of bedding for indoor ducks outside, and their phylogenetic proximity to wild ducks. This pattern is not fully backed up by the challenge studies, which typically find that the susceptibility of ducks is highly strain specific (Aldous et al., 2010; Spackman et al., 2010). However, their lack of clinical signs even when infected (Pillai et al., 2010) may allow for undetected circulation in domestic duck farms (Verhagen et al., 2021). As such, these domestic duck farms may allow for circulation and mutation, especially of LPAI strains to HPAI strains, before then passing it along to another poultry farm (Verhagen et al., 2021).

Indoor geese

We have no direct measure for this as geese are rarely studied, and when included they are typically pooled with ducks. Hill et al., (2019), reused the value from ducks. We have no additional information to adjust the metric, but perhaps it could be shifted down some since we know that wild geese at least are less likely to be infected than wild dabblers (Kent et al., 2022). For our model purposes, we do not use geese in subsequent analyses.

Outdoor layers/breeders

It is clear from the literature that outdoor birds are at greater risk than indoor birds as there is less biosecurity preventing either direct contact with wild birds or from aerosolized viruses. In addition to both Gonzales et al., (2012) and Bouwstra et al., (2017), finding greater risk for outdoor chickens than indoor chickens (11.1 and 6.3, respectively), in Italy, Galletti et al., (2018), found a strongly significant increase in risk for birds that are kept outdoors, though this paper does not include a species interaction.

Outdoor broiler

There is no information on outdoor broilers, as at least in Europe these are generally kept inside. The same is generally true for the United States, though free range, pasture fed, and organic birds may be allowed access to the outside. It is likely they should be at a lower risk for commercial birds than layers/breeders for the same reasons as indoor birds, i.e., a shorter lifespan. However, this is likely not true for backyard birds, as these are generally kept for a longer time and harvested slowly as needed. As such, we revised this down for commercial birds (11.1*0.2=2.2) and maintained it as the same for layers/breeders at 11.1.

Outdoor turkeys

As most European turkeys are housed inside, there is not a good value given for outdoor turkeys. Similarly, the bulk of U.S. turkey production has been moved indoors in response to what used to be yearly mass LPAI outbreaks in the Midwest on outdoor turkey farms (personal communication, Patyk). However, organic, pasture raised, and free-range birds are kept outdoors. Hill et al., (2019), use the value for chickens, though this is almost certainly an underestimate, as indoor turkeys are clearly at an increased risk compared to indoor chickens likely because of greater susceptibility to infection from AIV (Aldous et al., 2010; Spackman et al., 2010). Likewise, they are likely at a lower risk than ducks for the same reasons as for indoor birds. As such, we calculated an average between the two.

Outdoor ducks

As currently weighted, this is the most at-risk poultry type. Of note, Gonzales et al., (2012) and Bouwstra et al., (2017) disagree on the ranking of outdoor vs. indoor ducks, with Bouwstra et al. (2017) finding that meat ducks, which are mostly kept indoors are at greater risk. However, these estimates have large confidence intervals and this trend is not significant. As such, we used the values reported in Gonzales et al., (2012) as well as the decision by Hill et al., (2019) because it was the more robust analysis and showed the expected trend.

Outdoor geese

As with indoor geese, direct values are not available. Hill et al., (2019) used the same value as for ducks, which might be an overestimate.

Game birds

There is little information available for game bird species, however they are not explicitly included in our models.

Poultry Risk Estimates- Hybrid model categories

Categories from the European studies do not neatly fall into the population categories for the United States (Table 4). Most notably for the U.S., we lack data on whether birds are housed inside. To account for this, we have matched each hybrid poultry model category to its relative risk for both indoor and outdoor birds from the European categories. Then in the next section we assign percentages of the number of birds of each group that are housed outdoors to calculate a weighted relative risk based on the percentage of birds housed outside. Table 4 includes the hybrid poultry model categories and their relative indoor and outdoor risks. Not all of these categories actually exist (e.g., backyard breeder turkeys) but we have calculated potential risk values to complete the matrix for potential future use. The values in this table come directly from Table 3 above.

Table 4. Poultry categories in the hybrid model.

Species	Commodity	Category	Sector	Indoor RR	Outdoor RR	Notes
Chicken	Broiler	Commercial	Production	0.2	2.2	
Chicken	Broiler	Commercial	Breeding	1	11.1	Treated as layer/breeder
Chicken	Layer	Commercial	Production	1	11.1	
Chicken	Layer	Commercial	Breeding	1	11.1	
Chicken	Layer pullets	Commercial	NA	1	11.1	
Turkey	All	Commercial	Production	7.7	18	
Turkey	All	Commercial	Breeding	7.7	18	
Chicken	Broiler	Backyard	Production	0.2	11.1	
Chicken	Broiler	Backyard	Breeding	1	11.1	Treated as layer/breeder
Chicken	Layer	Backyard	Production	1	11.1	
Chicken	Layer	Backyard	Breeding	1	11.1	
Chicken	Layer pullets	Backyard	NA	1	11.1	
Turkey	All	Backyard	Production	7.7	18	
Turkey	All	Backyard	Breeding	7.7	18	

Percent of commercial poultry housed outside

There are three categories of commercial poultry that can be housed outside. First, birds labeled as “organic” must be allowed access to the outside for at least half of their lifetime. Second, birds labeled as “free range” must meet the same outdoor access requirements as organic, but simply don’t need to be raised in an organic manner. That is to say, these are not mutually exclusive categories where a bird that is organic is also free range, and a free-range bird can, but does not need to be, organic. Third, some birds are sold as “pasture raised.” This term is not regulated by the USDA and there are no specific housing requirements for a bird to be pasture raised. Due to data availability, we are only able to quantify the number of organic farms. The term “pasture raised” is used for marketing but does not have guidelines or underlying regulation. As such, we are unable to quantify this, and assume that the numbers are negligible.

To get estimates for the percentage of birds that are organic, we have combined the 2019 organic farm survey data available in Table 17 (organics_1_017 _017.pdf (usda.gov)) which gives the number of farms that have organic birds with the 2017 USDA, NASS Census of Agriculture (CoA) to get a total number of farms. These are part of the 2017 CoA. Table 5 summarizes these data and contains places to include data on free range or pasture raised birds if the data becomes available. The percentage of farms with organic poultry is the number of

organic farms divided by the total number of farms. Note that this is not actually the percentage of organic farms or percentage of poultry raised organically, but the percentage of farms that have any organic birds raised out of their total flock. Lastly, we have done this for the extent of the United States but can break these data out by state, if desired.

Table 5. Housing of commercial poultry.

Species	Type	#OrganicFarms	#FreeRangeFarms	#PastureRaised	#TotalFarms	Percentage
Chicken	Broiler	369	NA	NA	42858	0.008609828
Chicken	Layer	1057	NA	NA	232500	0.004546237
Turkey	All	129	NA	NA	23173	0.005566823

Percent of backyard poultry housed outside

We used an available dataset on backyard poultry included in a 2004 survey from the USDA on backyard poultry practices (Cover (usda.gov)). Documentation of the original USDA survey questionnaire is publicly available. This study surveyed people with backyard poultry in 18 major poultry producing states. Within these states, they identified 350 large commercial poultry operations with >10,000 chickens or 5,000 turkeys. Then, animal health officials canvassed the area within a 1-mile radius of these farms for residents with backyard birds (defined as no more than 1,000 birds) on their properties, excluding pets. Residents with birds were then asked to complete the questionnaire. Hence, these are not a random sample of all backyard birds, but instead a sample of backyard birds located next to major commercial poultry operations within major poultry producing states.

The most relevant information at hand is found in section I.B.3. Residents with backyard flocks were asked if they had any poultry on site that fit into any of three categories: Outdoors confined to property, outdoors able to leave property, or inside. Again, this was a "check all that apply" question, and many residents checked multiple options (as these raw totals add up to ~170%). In addition, those that had poultry housed inside (i.e., a barn or coop) were asked if they were allowed to go outside, of which a total of 65% of residents with indoor birds said "yes". Thus, 65% of the 73% of farms with indoor poultry allow these indoor poultry to go outside.

This type of survey data leads to a problem in trying to estimate either the number of birds housed outside or the number of residents with birds outside as residents could check more than one box. We calculated overall percentages using two methods. The first re-weights the data to calculate the percentage outdoors. If residents are equally likely to check any combination of boxes (an assumption that is likely not true), this would give a reasonable value. This is the sum of percent of birds allowed off property, confined to property, and inside birds times % allowed outside, divided by the sum of the three percentages. This is the value included in the next table. We have also calculated a minimum number that are allowed outside, which assumes that all of the farms with indoor poultry only had indoor poultry. This is calculated as one minus the percentage of residents that had inside poultry times the proportion of inside poultry not allowed outside. This represents the lowest possible percentage of outdoor birds. We have also created a maximum estimate for the number of birds kept outside, which assumes that no residents had birds that were both outside and allowed off property as well as outside and confined to property,

and that their number of inside birds was negligible in comparison to the number of outside birds.

In addition, those that had poultry housed inside (i.e., a barn or coop) were asked if they were allowed to go outside, of which a total of 65% of residents with indoor birds said “yes”.

The following table summarizes this data. We have calculated this for the overall data but could also go back and get separate estimates for the Southwest, Midwest, East, and Southeast regions. This data is not broken down by poultry type.

Table 6. Housing of backyard birds.

Off Property	On Property	Inside	%Inside Allowed Out	Base Percent	Min Outside	Max Outside
0.471	0.515	0.733	0.65	0.851	0.74345	0.996262

Obtaining relative risk values

The weights from these tables then go into the table of hybrid model poultry types and are used to calculate a weighted risk that takes into account the percentage of birds housed outdoors.

Table 7. Relative risk by poultry types in the hybrid model.

Species	Commodity	Category	Sector	IndoorRR	OutdoorRR	%Outdoor	WeightedRR
Chicken	Broiler	Commercial	Production	0.2	2.2	0.00860983	0.217219656
Chicken	Broiler	Commercial	Breeding	1	11.1	0	1
Chicken	Layer	Commercial	Production	1	11.1	0.00454624	1.045916989
Chicken	Layer	Commercial	Breeding	1	11.1	0	1
Chicken	Layer pullets	Commercial	NA	1	11.1	0	1
Turkey	All	Commercial	Production	7.7	18	0.00556682	7.757338282
Turkey	All	Commercial	Breeding	7.7	18	0	7.7
Chicken	Broiler	Backyard	Production	0.2	11.1	0.85075625	9.473243165
Chicken	Broiler	Backyard	Breeding	1	11.1	0.85075625	9.592638162
Chicken	Layer	Backyard	Production	1	11.1	0.85075625	9.592638162
Chicken	Layer	Backyard	Breeding	1	11.1	0.85075625	9.592638162
Chicken	Layer pullets	Backyard	NA	1	11.1	0.85075625	9.592638162
Turkey	All	Backyard	Production	7.7	18	0.85075625	16.46278941
Turkey	All	Backyard	Breeding	7.7	18	0.85075625	16.46278941

There are a few important things to note.

1. We currently lack data on free range and pasture raised poultry that are not certified organic. We are currently assuming that these numbers are negligible.
2. We assume that breeders for all commercial types are only housed indoors, as there is not a cost benefit to the farms for raising these organically/outdoors.

3. We do not have data on layer pullets being kept indoors/outdoors. Currently we are assuming that commercial birds are also always kept indoors while backyard birds are housed outdoors at the same proportions as for other poultry types.
4. All backyard poultry types have the same percentage of birds kept outdoors because we cannot break this data down by poultry type.
5. Breeders of any type are treated as layer/breeder for their relative risk estimates. There may be greater biosecurity at breeder facilities, however we currently have no way to quantify this. That said, the assumption that all breeders are kept indoors does account for this somewhat by lowering the estimate compared to the other species and production types.
6. Currently we are using different values for commercial and backyard broilers, where outdoor commercial broilers are given a lower relative risk because of their short lifespan. However, this is probably less true for backyard broilers, so they are instead assigned the same relative risks as layer/breeders. It is possible that we should also do this for indoor backyard broilers, but we have currently left them with the standard indoor broiler relative risk.

Literature Cited

Aldous, E. W., Seekings, J. M., McNally, A., Nili, H., Fuller, C. M., Irvine, R. M., Alexander, D. J., & Brown, I. H. (2010). Infection dynamics of highly pathogenic avian influenza and virulent avian paramyxovirus type 1 viruses in chickens, turkeys and ducks. *Avian Pathology*, *39*(4), 265–273. https://doi.org/10.1080/03079457.2010.492825

Bouwstra, R., Gonzales, J. L., de Wit, S., Stahl, J., Fouchier, R. A. M., & Elbers, A. R. W. (2017). Risk for low pathogenicity avian influenza virus on poultry farms, The Netherlands, 2007–2013. *Emerging Infectious Diseases*, *23*(9), 1510–1516. https://doi.org/10.3201/eid2309.170276

Galletti, G., Santi, A., Guberti, V., Paternoster, G., Licata, E., Loli Piccolomini, L., Procopio, A., & Tamba, M. (2018). A method to identify the areas at risk for the introduction of Avian Influenza virus into poultry flocks through direct contact with wild ducks. *Transboundary and Emerging Diseases*, *65*(4), 1033–1038. https://doi.org/10.1111/tbed.12838

Gonzales, J. L., Elbers, A. R. W., Bouma, A., Koch, G., de Wit, J. J., & Stegeman, J. A. (2010). Low-pathogenic notifiable avian influenza serosurveillance and the risk of infection in poultry - a critical review of the European Union active surveillance programme (2005-2007). *Influenza and Other Respiratory Viruses*, *4*(2), 91–99. https://doi.org/10.1111/j.1750-2659.2009.00126.x

Gonzales, J. L., Stegeman, J. A., Koch, G., de Wit, S. J., & Elbers, A. R. W. (2012). Rate of introduction of a low pathogenic avian influenza virus infection in different poultry production sectors in the Netherlands. *Influenza and Other Respiratory Viruses*, *7*(1), 6–10. https://doi.org/10.1111/j.1750-2659.2012.00348.x

Hill, A., Gillings, S., Berriman, A., Brouwer, A., Breed, A. C., Snow, L., Ashton, A., Byrne, C., & Irvine, R. M. (2019). Quantifying the spatial risk of Avian Influenza introduction into British poultry by wild birds. *Scientific Reports*, *9*(19973). https://doi.org/10.1038/s41598-019-56165-9 5

Kent, C. M., Ramy, A. M., Ackerman, J. T., Bahl, J., Bevins, S. N., Bowman, A. S., Boyce, W., Cardona, C., Casazza, M. L., Cline, T. D., De La Cruz, S. E., Hall, J. S., Hill, N. J., Ip, H., Krauss, S., Mullinax, J. M., Nolting, J. M., Plancarte, Ma., Poulson, R., … Prosser, D. J. (2022). Spatiotemporal changes in influenza A virus prevalence among wild waterfowl inhabiting the continental United States throughout the annual cycle. *Scientific Reports, 12*(13083).

Mutinelli, F., Capua, I., Terregino, C., & Cattoli, G. (2003). Clinical, gross, and microscopic findings in different avian species naturally infected during the H7N1 low- and high-pathogenicity avian influenza epidemics in Italy during 1999 and 2000. *Avian Diseases*, *47*(SPEC. ISS.), 844–848. https://doi.org/10.1637/0005-2086-47.s3.844

Pantin-Jackwood, M. J., Stephens, C. B., Bertran, K., Swayne, D. E., & Spackman, E. (2017). The pathogenesis of H7N8 low and highly pathogenic avian influenza viruses from the United States 2016 outbreak in chickens, turkeys and mallards. *PLoS ONE*. https://doi.org/10.1371/journal.pone.0177265

Pillai, S. P. S., Pantin-Jackwood, M., Yassine, H. M., Saif, Y. M., & Lee, C. W. (2010). The high susceptibility of turkeys to influenza viruses of different origins implies their importance as potential intermediate hosts. *Avian Diseases*, *54*, 522–526.

Spackman, E., Gelb, J., Preskenis, L. A., Ladman, B. S., Pope, C. R., Pantin-Jackwood, M. J., Mckinley, E. T., Gelb Jr., J., Preskenis, L. A., Ladman, B. S., Pope, C. R., Pantin-Jackwood, M. J., & Mckinley, E. T. (2010). The pathogenesis of low pathogenicity H7 avian influenza viruses in chickens, ducks and turkeys. *Virology Journal*, *7*(331), 1–10. https://doi.org/10.1186/1743-422X-7-331

Verhagen, J. H., Fouchier, R. A. M., & Lewis, N. (2021). Highly Pathogenic Avian Influenza Viruses at the Wild-Domestic Bird Interface in Europe: Future Directions for Research and Surveillance. *Viruses*, *13*(2). https://doi.org/10.3390/v13020212
